# Blockade-of-Binding Activities toward Envelope-Associated, Type-Specific Epitopes as a Correlative Marker for Dengue Virus-Neutralizing Antibody

**DOI:** 10.1128/spectrum.00918-23

**Published:** 2023-07-06

**Authors:** Poonsook Keelapang, Romchat Kraivong, Rojjanaporn Pulmanausahakul, Rungtawan Sriburi, Eakachai Prompetchara, Jutamart Kaewmaneephong, Nicha Charoensri, Pattarakul Pakchotanon, Thaneeya Duangchinda, Piyanan Suparattanagool, Prasit Luangaram, Promsin Masrinoul, Juthathip Mongkolsapaya, Gavin Screaton, Kiat Ruxrungtham, Prasert Auewarakul, Sutee Yoksan, Prida Malasit, Chunya Puttikhunt, Chutitorn Ketloy, Nopporn Sittisombut

**Affiliations:** a Department of Microbiology, Faculty of Medicine, Chiang Mai University, Chiang Mai, Thailand; b Medical Biotechnology Research Unit, BIOTEC, NSTDA, Bangkok, Thailand; c Molecular Biology of Dengue and Flaviviruses Research Team, Medical Molecular Biotechnology Research Group, National Center for Genetic Engineering and Biotechnology (BIOTEC), National Science and Technology Development Agency (NSTDA), Pathumthani, Thailand; d Division of Dengue Hemorrhagic Fever Research, Faculty of Medicine, Siriraj Hospital, Mahidol University, Bangkok, Thailand; e Siriraj Center of Research Excellence in Dengue and Emerging Pathogens, Faculty of Medicine, Siriraj Hospital, Mahidol University, Bangkok, Thailand; f Princess Srisavangavadhana College of Medicine, Chulabhorn Royal Academy, Bangkok, Thailand; g Center of Excellence in Vaccine Research and Development (Chula-VRC), Faculty of Medicine, Chulalongkorn University, Bangkok, Thailand; h Department of Laboratory Medicine, Faculty of Medicine, Chulalongkorn University, Bangkok, Thailand; i Center for Research and Development of Medical Diagnostic Laboratories, Faculty of Associated Medical Sciences, Khon Kaen University, Khon Kaen, Thailand; j Clinical Epidemiology Unit, Faculty of Medicine, Khon Kaen University, Khon Kaen, Thailand; k Center for Vaccine Development, Institute of Molecular Biosciences, Mahidol University at Salaya, Nakhon Pathom, Thailand; l Wellcome Centre for Human Genetics, Nuffield Department of Medicine, University of Oxford, Oxford, United Kingdom; m Chinese Academy of Medical Science (CAMS), Oxford Institute (COI), University of Oxford, Oxford, United Kingdom; n Department of Microbiology, Faculty of Medicine, Siriraj Hospital, Mahidol University, Bangkok, Thailand; Centro de Investigacion y de Estudios Avanzados del Instituto Politecnico Nacional

**Keywords:** dengue virus, blockade of binding, ELISA, neutralizing antibody

## Abstract

Humans infected with dengue virus (DENV) acquire long-term protection against the infecting serotype, whereas cross-protection against other serotypes is short-lived. Long-term protection induced by low levels of type-specific neutralizing antibodies can be assessed using the virus-neutralizing antibody test. However, this test is laborious and time-consuming. In this study, a blockade-of-binding enzyme-linked immunoassay was developed to assess antibody activity by using a set of neutralizing anti-E monoclonal antibodies and blood samples from dengue virus-infected or -immunized macaques. Diluted blood samples were incubated with plate-bound dengue virus particles before the addition of an enzyme-conjugated antibody specific to the epitope of interest. Based on blocking reference curves constructed using autologous purified antibodies, sample blocking activity was determined as the relative concentration of unconjugated antibody that resulted in the same percent signal reduction. In separate DENV-1-, -2-, -3-, and -4-related sets of samples, moderate to strong correlations of the blocking activity with neutralizing antibody titers were found with the four type-specific antibodies 1F4, 3H5, 8A1, and 5H2, respectively. Significant correlations were observed for single samples taken 1 month after infection as well as samples drawn before and at various time points after infection/immunization. Similar testing using a cross-reactive EDE-1 antibody revealed a moderate correlation between the blocking activity and the neutralizing antibody titer only for the DENV-2-related set. The potential usefulness of the blockade-of-binding activity as a correlative marker of neutralizing antibodies against dengue viruses needs to be validated in humans.

**IMPORTANCE** This study describes a blockade-of-binding assay for the determination of antibodies that recognize a selected set of serotype-specific or group-reactive epitopes in the envelope of dengue virus. By employing blood samples collected from dengue virus-infected or -immunized macaques, moderate to strong correlations of the epitope-blocking activities with the virus-neutralizing antibody titers were observed with serotype-specific blocking activities for each of the four dengue serotypes. This simple, rapid, and less laborious method should be useful for the evaluation of antibody responses to dengue virus infection and may serve as, or be a component of, an *in vitro* correlate of protection against dengue in the future.

## INTRODUCTION

Dengue viruses (DENVs) are mosquito-borne, enveloped, positive-stranded RNA viruses that belong to the family *Flaviviridae*. Dengue virus infection causes diseases such as undifferentiated fever, dengue fever, dengue hemorrhagic fever, and unusual manifestations (1). Dengue continues to be an important public health problem for tropical countries; approximately 58 million disease cases were reported in 2013 alone (2). Most dengue virus-infected individuals are asymptomatic, but some exhibit delayed viral clearance and serve as sources for further transmission (3). Two live-attenuated tetravalent vaccine candidates (Dengvaxia and TAK-003) have been tested in phase III clinical trials, but neither has shown strong protective efficacy against all four dengue virus serotypes (4). The protection provided by Sanofi Pasteur’s Dengvaxia vaccine lasts for about 3 years after the third dose (5).

With a few exceptions (6, 7), dengue virus infection induces long-term protection against the infecting (homologous) serotype; however, cross-protection against other serotypes is short-lived (8). In primarily infected persons, a considerable proportion of virion-binding antibodies target the viral premembrane (prM) protein of multiple serotypes and are not, or are only weakly, neutralizing (9, 10). Targets of virus-neutralizing antibodies reside on the major envelope protein, E, which consists of distinct structural domains, including the central E domain I (EDI) domain, the elongated EDII domain involved in fusion, and the receptor-binding globular EDIII domain (11). Sequence variations in the E protein result in serotype-specific epitopes; common epitopes found in two, three, or all four dengue serotypes; and epitopes that are shared with other flaviviruses. Depending on the maturation state of a virion, the E protein may exist as a prM-E heterodimer and/or an E-E homodimer, generating quaternary-structure-dependent epitopes in addition to the tertiary-structure-associated epitopes present in monomeric envelope protein molecules (11, 12). Most antibodies recognizing the receptor-binding/fusion E protein are cross-reactive fusion-loop-binding antibodies, which can be adsorbed with noninfecting (heterologous) serotypes; however, only a small fraction are type specific (13). Neutralization of the homologous serotype is mediated mainly by type-specific antibodies, many of which recognize quaternary-structure-dependent epitopes (14, 15). Neutralizing antibody activities against heterologous serotypes are weak or absent, and cross-reactive E-binding antibodies do not appear to contribute to neutralization (13).

Humoral immune responses in secondary infections predominantly comprise high-avidity and potently neutralizing cross-reactive antibodies and cross-reactive memory cell-derived plasmablasts (16–20). The proportion of antibodies recognizing epitopes present in the monomeric E protein increases in secondary infections (21). Based on data from several studies, the proportion of type-specific and cross-reactive monoclonal antibodies generated after primary DENV infection is estimated to be 23 versus 77% (22). Following secondary DENV infection, this proportion drastically changes to 3 versus 97% (22). The immune responses following a secondary infection are diverse as some individuals have predominantly cross-reactive neutralizing antibodies in their sera, while in others, both type-specific and cross-reactive antibodies contribute to virus neutralization (21). Only type-specific antibodies against previously encountered serotypes are found in secondary infections (21).

Since its introduction in 1967 (23), the plaque reduction neutralization test (PRNT) has been valuable for studying the antibody-mediated response to dengue virus infection and monitoring the effect of vaccination (5, 24–28). This test is laborious, has low throughput, lacks standardization, and demonstrates high variability, which limits its usage (24, 29–33). Furthermore, the PRNT is an endpoint assay of neutralizing antibodies that is defined as the highest dilution at which a certain percent threshold of plaque number reduction, such as 50%, 75%, 80%, or 90%, is reached. Because of the dilution, the PRNT may not reflect *in vivo* neutralization in which pathogens encounter a myriad of antibodies present at various concentrations in undiluted plasma. Potential interactions between antibodies occupying adjacent sites or those with distinct functions and effects may also be reduced as a consequence of dilution. Moreover, the use of a small number of infectious viruses as the target of antibodies precludes the detection of plaque-reducing activity through mechanisms requiring a large number of viral particles, such as aggregation (34). Several variants of the PRNT and alternative assays have been proposed (35–38), but none have been tested extensively.

In this study, we explored whether a blockade-of-binding type of assay (15, 39–42), which provides information on antibodies that recognize an epitope of interest on a viral particle, would be useful as an *in vitro* correlative marker of neutralizing antibodies against dengue virus. Blood specimens were obtained from macaques infected with dengue virus or immunized with different prime-boost vaccination approaches. For each dengue virus serotype-related set of samples, the correlation between the blockade-of-binding activity and the neutralizing antibody was assessed using selected type-specific and cross-reactive neutralizing monoclonal antibodies with known binding sites (12).

## RESULTS AND DISCUSSION

### Correlation between type-specific epitope-blocking activity and virus-neutralizing titers 1 month after dengue virus infection.

**FIG 1:**
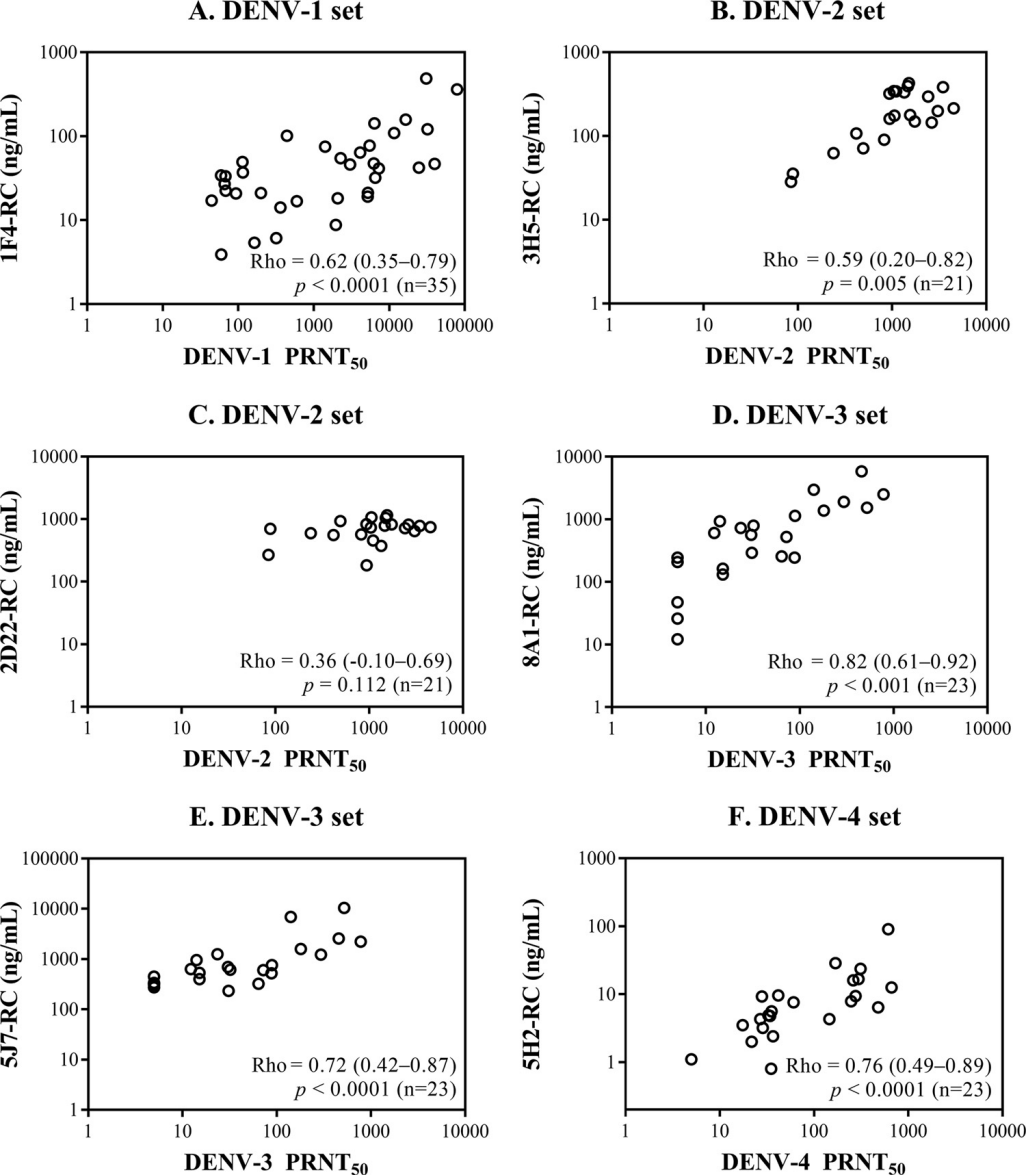
Relationship between the neutralizing antibody titer and the type-specific epitope-blocking activity in macaque blood samples taken 30 days after infection with recent clinical isolates or attenuated strains. Scatterplots show the neutralizing antibody titers (PRNT_50_) and type-specific epitope-blocking activities (RC [relative concentration]) on a log_10_ scale to facilitate the visual inspection of all values. The Spearman correlation coefficient (rho) derived from nontransformed data is shown on each plot together with the 95% confidence interval in parentheses, the *P* value, and the number of macaques (n).

The blocking activities toward one or two dengue virus type-specific epitopes were determined for each serotype in macaque sera 30 days after virus infection and plotted against the corresponding neutralizing antibody activities (Fig. 1). The correlation between the DENV-1 PRNT_50_ (PRNT with a 50% reduction endpoint) and the 1F4 epitope-blocking activity was significant for the DENV-1 serum set (Fig. 1A). For the DENV-2 serum set, the correlation between the DENV-2 PRNT_50_ and the 3H5 epitope-blocking activity was significant (Fig. 1B), whereas the correlation with the 2D22 epitope-blocking activity was not significant (Fig. 1C). Because of the weak binding of 2D22 to the prototypic DENV-2 strain 16681 in preliminary tests, strain 16681-prE203A (a prM junction mutant with enriched mature extracellular particles as a consequence of enhanced prM cleavage [43]) replaced strain 16681 in all 2D22 epitope-blocking experiments. The weak and statistically nonsignificant correlation between the 2D22 epitope-blocking activity and the DENV-2 PRNT_50_ was consistent with the low levels of 2D22 epitope-blocking activity found in DENV-2-infected macaques as well as those infected with other serotypes (see Fig. S2 in the supplemental material), suggesting that cross-reacting antibodies may contribute to the observed 2D22 epitope-blocking activity in DENV-2-infected macaques. For the DENV-3 and DENV-4 serum sets, although the neutralizing antibody activity remained undetectable in some macaques after infection with DENV-3 or -4 strains, the epitope-blocking activity was always detectable, likely as a result of the use of the autologous-blocking reference curve for the calculation of blocking activity. Nevertheless, significant correlations were observed between the blocking activities of two anti-DENV-3 antibodies (Fig. 1D and E) and an anti-DENV-4 antibody (Fig. 1F) and the corresponding viral serotype PRNT_50_.

### Correlation between changes in type-specific epitope-blocking activity and virus-neutralizing titers in pooled samples.

The lack of a correlation between the 2D22 epitope-blocking activity and the DENV-2 PRNT_50_ observed 1 month after infection may also reflect the high variation in 2D22 epitope-blocking levels among macaques infected with serotypes other than DENV-2 (Fig. S2). To account for variation among individuals, blood samples drawn from each macaque before and at various time points after infection or immunization were used for testing, and the correlation of the epitope-blocking activity and the neutralizing antibody titer in each pooled serum set was determined by repeated-measures correlation.

### (i) DENV-1.

**FIG 2:**
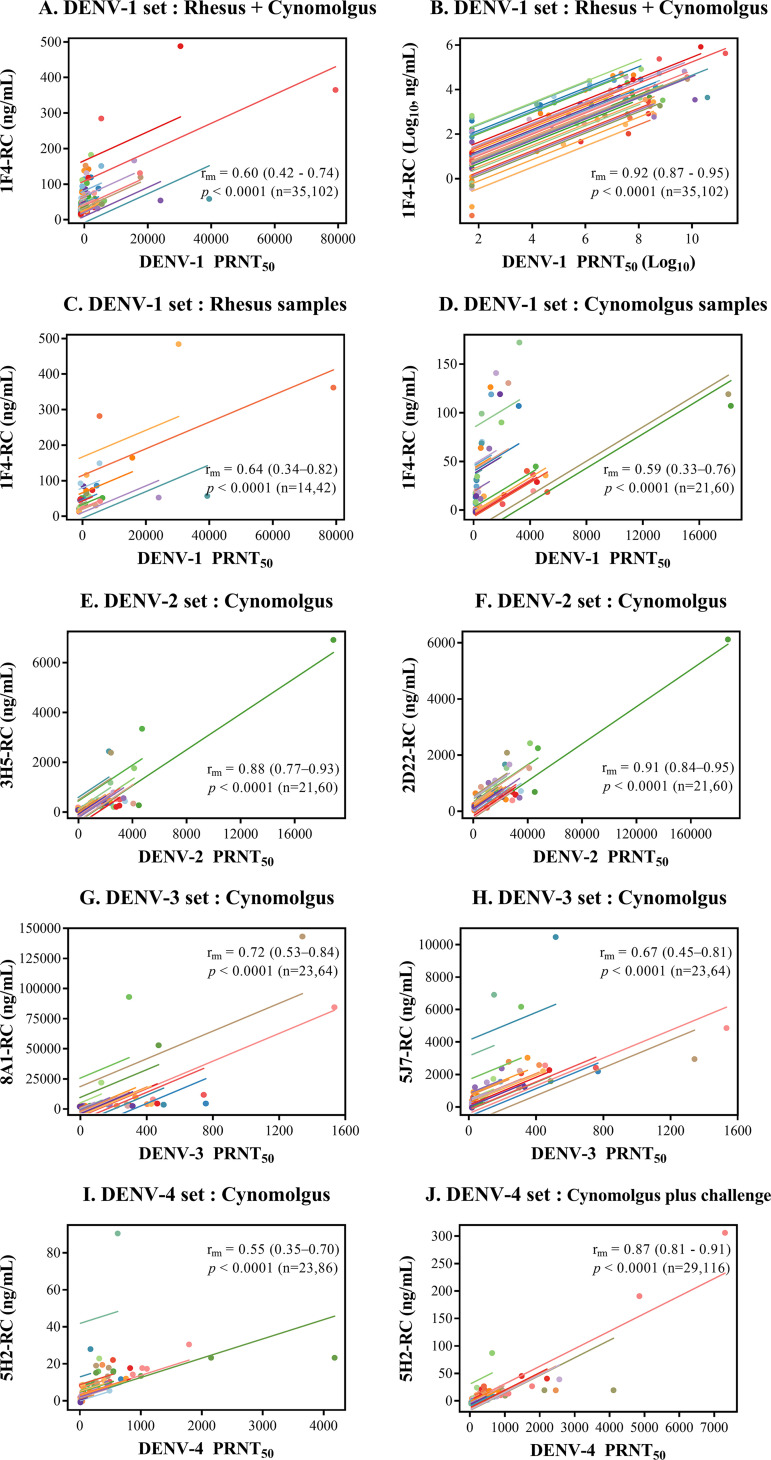
Relationship between the neutralizing antibody titer and the type-specific epitope-blocking activity in pooled blood samples. Scatterplots show the relationship between the neutralizing antibody titer (PRNT_50_) and the type-specific epitope-blocking activity (RC [relative concentration]). Colored lines represent regression lines for each macaque. The repeated-measures correlation coefficient (*r*_rm_) is shown on each plot together with the 95% confidence interval in parentheses, the *P* value, and the numbers of macaques and blood samples (n), respectively.

The 1F4 epitope-blocking activity was initially assessed in a set of 102 rhesus and cynomolgus macaque blood samples collected within 30 days of DENV-1 infection or DENV-1-related immunization injections (see Table 2). The repeated-measures correlation between the 1F4 epitope-blocking activity and the DENV-1 PRNT_50_ (Fig. 2A) was significant, which is consistent with the results of the experiment using one sample per macaque (Fig. 1A), as described above. In the analysis of the pooled samples, the range of values was so wide that low values and individual regression lines could not be depicted clearly on a linear scale. When the data were log transformed and analyzed in the same manner, the graphical representation was improved (Fig. 2B). However, fitting the log-transformed data into the rmcorr model resulted in an inflated correlation coefficient (repeated-measures correlation coefficient [*r*_rm_] = 0.92) (Fig. 2B), which, due to a reduction in differences among repeated samples secondary to the transformation and the resultant better fit of the data points to the regression, likely represented an overestimation of the strength of the correlation. Subsequent analyses were performed using nontransformed data.

When the relationship between the 1F4 epitope-blocking activity and the DENV-1 PRNT_50_ was determined separately for each monkey species, similar levels of correlation were observed for samples from rhesus macaques and cynomolgus macaques (Fig. 2C and D).

### (ii) DENV-2 and DENV-3.

Due to geographical isolation, a rhesus macaque was not available at the Indonesian animal facility employed for most non-DENV-1-related investigations. Further studies were performed using only cynomolgus macaque blood samples. In contrast to the moderate (Fig. 1B) and weak (Fig. 1C) Spearman correlations, strong repeated-measures correlations were observed for the neutralizing activity against DENV-2 and the blocking activity for the two serotype 2-specific antibodies (Fig. 2E and F). Significant repeated-measures correlations were observed between DENV-3 PRNT_50_ values and both 8A1 epitope- and 5J7 epitope-blocking activities (Fig. 2G and H). However, the correlations were not markedly stronger than the Spearman correlations, which did not account for individual variations (Fig. 1B to E). These findings suggest that variation among individuals has a greater effect on the relationship between type-specific epitope-blocking activity and neutralizing activity in the context of DENV-2 infection/immunization than in the cases of DENV-1 and DENV-3.

A comparison of the 3H5 and 2D22 epitope-blocking activities present in macaques that had been infected with non-DENV-2 strains revealed higher levels of 2D22 epitope-blocking activities among four of the eight DENV-4-infected macaques than in the DENV-2-infected group (Fig. S2). Among DENV-3-infected macaques, low 5J7 epitope-blocking activities below the baseline for serotype-specific detection were detected in the majority (3/5) (Fig. S2). This finding is consistent with the results of a recent study in human volunteers who received the tetravalent live-attenuated dengue TV003 vaccine, suggesting that the 5J7 epitope is not the major target of DENV-3 type-specific neutralizing antibodies (44). Based on these limited findings, 3H5 and 8A1 may be the preferred choices for use in blockade-of-binding assays to measure serotype-specific antibodies against DENV-2 and DENV-3, respectively.

### (iii) DENV-4.

The relationship between the PRNT_50_ against DENV-4 and the 5H2 epitope-blocking activity was examined in blood samples taken from 23 cynomolgus macaques captured at two locations (Indonesia and Thailand), with a difference in the long interval between the priming and boosting injections in the Thailand setting (Table S1). There was a significant but moderate correlation for a set of 86 samples collected at various time points spanning the period between the preinjection day and day 118 or 270 postinjection, which represented day 30 after the second boost in these live-attenuated vaccine (LAV)-primed macaques (Fig. 2I). When 30 additional samples collected 2 and/or 4 weeks after live-virus challenge with a recent DENV-4 isolate were included in the analysis, the correlation was markedly stronger (Fig. 2J).

### Correlation between common epitope-blocking activity and neutralizing antibody titers.

**FIG 3:**
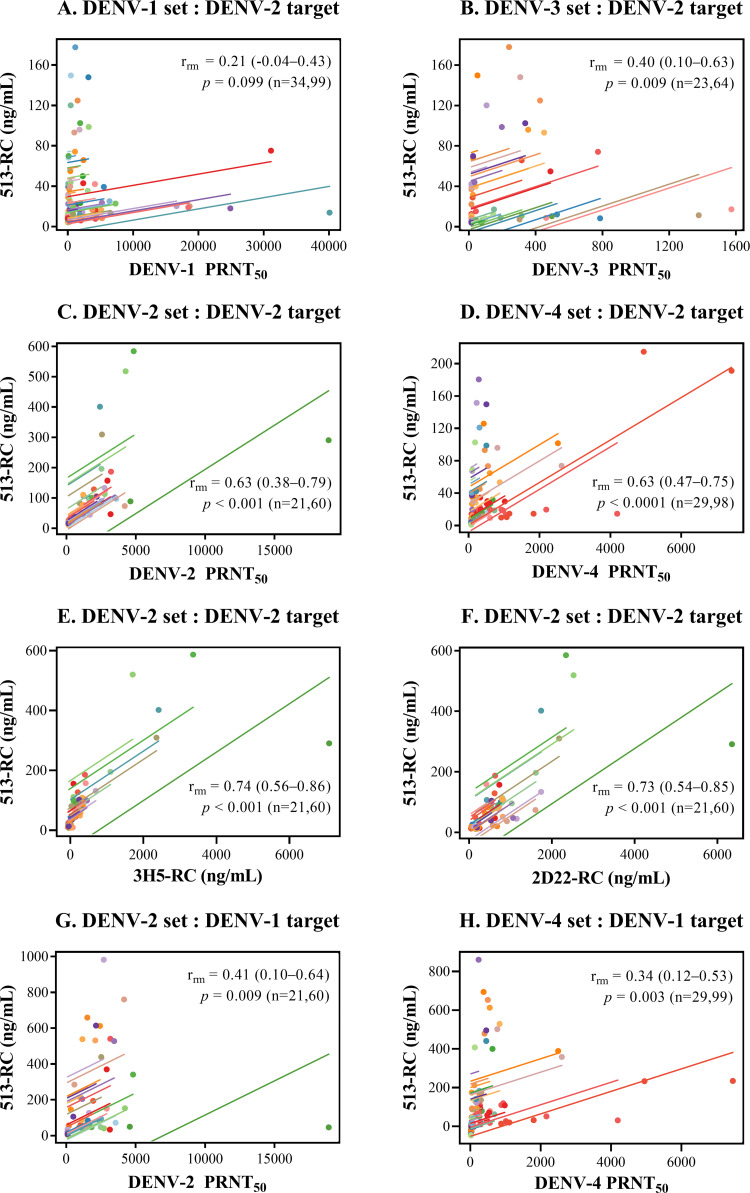
Relationship between the neutralizing antibody titer, the 513 epitope-blocking activity, and DENV-2-specific epitope-blocking activities in pooled blood samples. Scatterplots and regression lines show the relationship between the 513 epitope-blocking activity and the neutralizing antibody titer (A to D, G, and H) and between the 513 epitope-blocking activity and DENV-2-specific epitope-blocking activities (E and F) for different sample sets. DENV-2 strain 16681 or prE203A was employed as a binding target in panels A to F; DENV-1 strain Hawaii was employed in panels G and H. The repeated-measures correlation coefficient (*r*_rm_) is shown on each plot together with the 95% confidence interval in parentheses, the *P* value, and the numbers of macaques and blood samples (n), respectively.

The relationship between the epitope-blocking activity targeting two epitopes on the DENV envelope protein common to all four serotypes (513 and EDE-1 C10) and the neutralizing antibody titer was initially assessed using DENV-2 particles as the target (Fig. 3).

### (i) 513 epitope.

In the combined set of rhesus and cynomolgus macaque samples, there was no significant correlation between the 513 epitope-blocking activity and the DENV-1 PRNT_50_ (Fig. 3A). The correlation with the DENV-3 PRNT_50_ was significant but weak (Fig. 3B). Significant and moderate correlations were observed between the 513 epitope-blocking activity and the DENV-2 PRNT_50_ and DENV-4 PRNT_50_ in the respective DENV-2- and DENV-4-related samples (Fig. 3C and D). The moderate correlation between the 513 epitope-blocking activities and the DENV-2 or DENV-4 PRNT_50_ may be explained by the interference of antibodies recognizing DENV-2-specific epitopes or DENV-2- and DENV-4-common epitopes present in the DENV-2-related and DENV-4-related sets of samples, respectively. The cross-reactivity between DENV-2 and DENV-4 is consistent with the observation that they are antigenically more similar to each other than to DENV-1 and DENV-3 strains (45).

Strong correlations were observed between the 513 epitope-blocking activity and the blocking activity against both of the DENV-2-specific antibodies (3H5 [Fig. 3E] and 2D22 [Fig. 3F]). The correlation of the 513 epitope-blocking activity with the 3H5 epitope-blocking activity in the DENV-2-related sample set agreed well with the direct cross-blocking effects observed between 513 and 3H5 (Fig. S1A and B), which can be explained by the proximity of the 513 and 3H5 epitopes in the EDIII domain of the DENV-2 particle (46, 47).

To better assess the extent to which the cross-serotype 513 epitope-binding activity of antibodies in DENV-2-related samples contributes to the DENV-2 PRNT_50_, DENV-1 particles were employed as targets. When excluding the signal of serotype 2-specific antibodies in this fashion, the correlation between the 513 epitope-blocking activity and the DENV-2 PRNT_50_ was markedly weaker (Fig. 3G), indicating that serotype 2-specific antibodies make a major contribution to the relationship between the 513 epitope-blocking activity and the DENV-2 PRNT_50_. A similar reduction in the correlation strength was observed for the DENV-4 PRNT_50_ and the 513 epitope-blocking activity when DENV-1 particles were employed as targets for DENV-4-related samples (Fig. 3H).

### (ii) C10 epitope.

**FIG 4:**
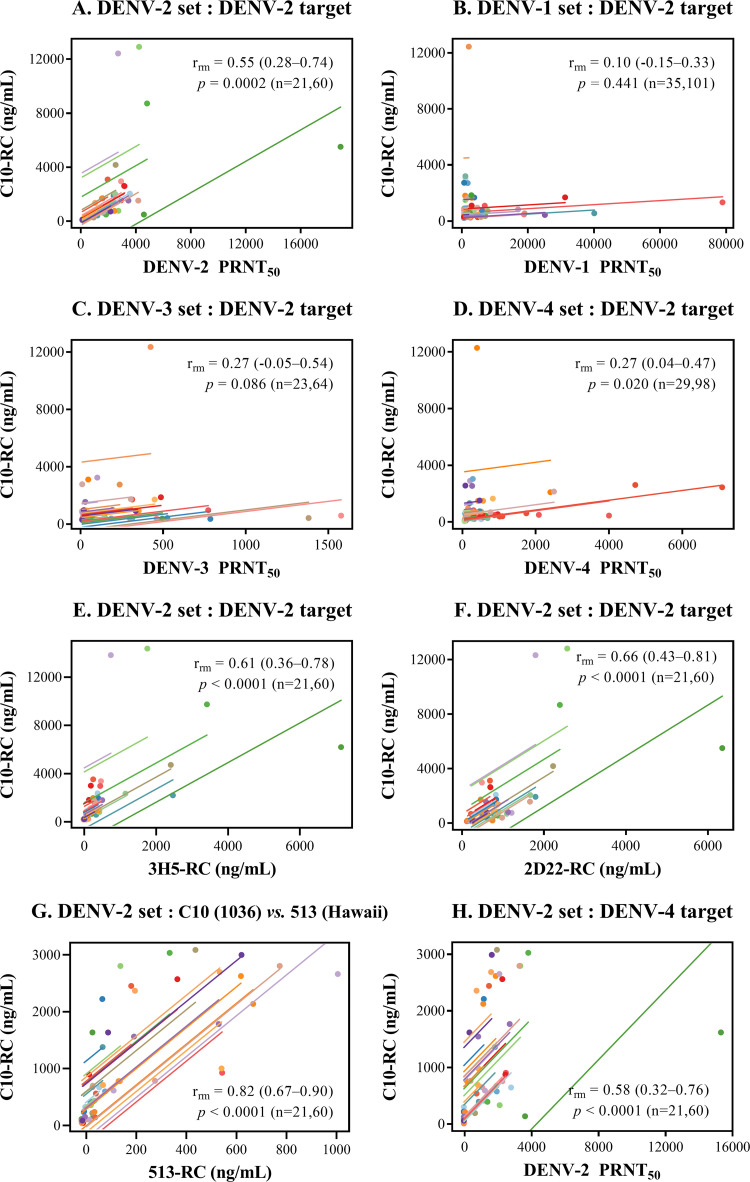
Relationship between the neutralizing antibody titer, the C10 epitope-blocking activity, and the DENV-2-specific epitope-blocking activity in macaque blood samples. Scatterplots and regression lines show the relationship between the C10 epitope-blocking activity and the neutralizing antibody titer (A to D and H) and between the C10 epitope-blocking activity and the DENV-2-specific epitope-blocking activity (E and F) or the 513 epitope-blocking activity (G) for different sample sets. DENV-2 strain 16681 or prE203A was employed as a binding target in panels A to G; a DENV-4 strain was used in panel H. The repeated-measures correlation coefficient (*r*) is shown on each plot together with the 95% confidence interval in parentheses, the *P* value, and the numbers of macaques and blood samples (n), respectively.

When the C10 epitope-blocking activity was assessed using DENV-2 particles as the target, a significant moderate correlation between the C10 epitope-blocking activity and the DENV-2 PRNT_50_ was observed in the DENV-2-related samples (Fig. 4A). The correlations between the C10 epitope-blocking activity and the DENV-1, -3, and -4 PRNT_50_ for the DENV-1-, -3-, and -4-related samples were weak and/or did not reach statistical significance (Fig. 4B to D). As in the case of the 513 epitope, moderate correlations were also observed between the C10 epitope-blocking activity and the epitope-blocking activities of 3H5 (Fig. 4E) and 2D22 (Fig. 4F) in the DENV-2-related sample set. These results suggest that the correlation between the DENV-2 PRNT_50_ and the C10 epitope-blocking activity may primarily reflect the blocking effect of DENV-2 serotype-specific antibodies. Further tests of the blocking activities in the DENV-2-related serum set were performed using non-DENV-2 viral particles to minimize the influence of DENV-2 type-specific antibodies. A strong correlation was observed between the C10 epitope-blocking activity (tested using DENV-4 particles) and the 513 epitope-blocking activity (tested using DENV-1 particles) (Fig. 4G). When DENV-4 particles were employed as the binding target, a significant but moderate correlation was observed between the DENV-2 PRNT_50_ and the C10 epitope-blocking activity, similar to that observed when DENV-2 particles were used (Fig. 4H), suggesting that in contrast to the 513 epitope-blocking activity, DENV-2 type-specific antibodies are not a major factor controlling the relationship between the C10 epitope-blocking activity and the DENV-2 PRNT_50_.

For DENV-2-related macaque samples, the correlations among the 3H5 epitope-, 2D22 epitope-, 513 epitope-, and C10 epitope-blocking activities were notable. These results suggest that infected/immunized macaque antibodies bind to overlapping regions on the DENV-2 envelope. This notion is supported by the cross-blocking effects of 3H5, 513, and 2D22 on C10 antibody binding (Fig. S1C). Intriguingly, the strong and very strong correlations between the DENV-2 PRNT_50_ and the 3H5 epitope- and 2D22 epitope-blocking activities (Fig. 2C and D, respectively) were in contrast to the moderate correlations with the 513 epitope- and C10 epitope-blocking activities (Fig. 3G and Fig. 4H, respectively). Antibodies binding to DENV-2-specific epitopes may therefore contribute to the neutralization of DENV-2 to a greater extent than those recognizing common epitopes. The important role of type-specific antibodies was demonstrated in recent phase III dengue vaccine trial studies showing that type-specific neutralizing antibodies are a better correlate of protection than the total level of neutralizing antibodies (44, 48, 49).

### (iii) Correlation of the PRNT_50_ with the DENV-2 particle-binding and EDIII-binding activities.

**FIG 5:**
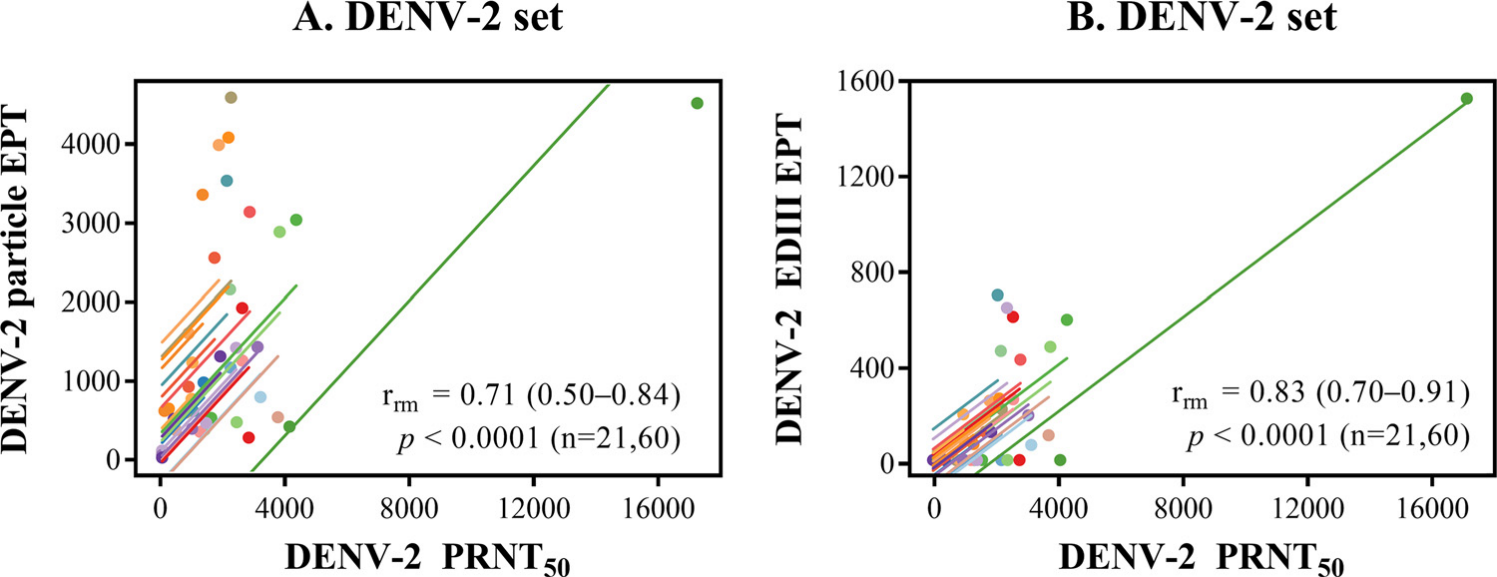
Relationship between the neutralizing antibody titer and binding activity in pooled blood samples. Scatterplots and regression lines show the relationship between the neutralizing antibody titer and the DENV-2 particle-binding activity (A) or the DENV-2 EDIII-binding activity (B) for the DENV-2 infection/immunization sample set. The repeated-measures correlation coefficient (*r*_rm_) is shown on each plot together with the 95% confidence interval in parentheses, the *P* value, and the numbers of macaques and blood samples (n), respectively. EPT, endpoint titer.

For DENV-2-related macaque samples, a significant and strong correlation was observed between the particle-binding titer and the DENV-2 PRNT_50_ (Fig. 5A), which was in the same range as the 3H5 epitope-blocking activity–DENV-2 PRNT_50_ correlation (Fig. 2E). The subtle difference in the *r*_rm_ values for these two correlations may reflect the finding that a large proportion of particle-binding antibodies are directed against prM, which does not result in strong neutralization (9, 10). By focusing on a limited set of critical envelope-antibody interactions and by competing out low-affinity antibodies with the selected monoclonal antibodies, the blockade-of-binding activity, particularly that directed at serotype-specific epitopes, may provide a more functionally relevant correlative marker of neutralization than the particle-binding activity.

It was shown previously that although the DENV-2 EDIII-specific IgG fraction formed a very small proportion of the antiviral antibody response in humans, it was significantly correlated with DENV-2 neutralization (50). For DENV-2-related macaque samples in this study, there was a strong correlation between the EDIII-binding IgG titer and the DENV-2 PRNT_50_ (Fig. 5B). However, when EDIII-binding activity testing was performed starting with a 1:40 dilution, it was not detected in three macaques 1 month after wild-type DENV-2 infection, and it was detected in only 1 out of 18 macaques after immunization with the DENV-2 attenuated strain (Table S2). In this case, the test for EDIII-binding antibodies appeared less sensitive than the epitope-blocking activity test.

In this study, we identified a set of type-specific antibodies suitable for the assessment of type-specific binding antibodies for the four dengue virus serotypes using a blockade-of-binding enzyme-linked immunosorbent assay (ELISA)-based approach. Our results showed moderate to strong correlations between the blockade-of-binding activities and the PRNT_50_. The limitations of this study are the small number of macaques/samples and the limited quantity of samples, which precluded the assessment of the antibody activities at a 1:20 dilution or lower. As we did not attempt to systematically search for the most discriminatory epitope for use in the measurement of type-specific antibodies and we were able to include only one or two serotype-specific epitopes per serotype, this may restrict the potential usefulness of testing persons infected with a viral variant that lacks the testing epitope, generating a false-negative result (31). The strengths of our study include the use of female macaques, the variety of ways in which the antibody response was induced and boosted, and the well-defined time points and duration of infection and immunization, which helped to delineate some of the factors affecting the relationship between the two testing parameters. The potential usefulness of the blockade-of-binding activity as a correlative marker of neutralizing antibodies against dengue viruses and as a correlate of protection needs to be further assessed in humans.

## MATERIALS AND METHODS

### Viruses.

Prototypic dengue viruses were provided by the late Ananda Nisalak of the Armed Forces Research Institute of Medical Sciences, Bangkok, Thailand, for use in neutralization tests and antibody binding (Table 1). Dengue virus strains were isolated from pediatric patients in Thailand between 2003 and 2005 (51). A DENV-2 prM junction mutant, strain 16681-prE203A (52), was used to determine the 2D22 epitope-blocking activity. Viruses were amplified in C6/36 cells using Leibovitz’s L15 maintenance medium (Invitrogen) supplemented with 1.5% (vol/vol) fetal bovine serum (HyClone) and 10% tryptose phosphate broth (Sigma) in ambient air at 29°C. As an exception, the DENV-1 Hawaii strain was amplified in Vero cells to obtain a high level of maturation for use in the 1F4 epitope-blocking assay. Vero cells were maintained in minimal essential medium (MEM) (Invitrogen) supplemented with 10% fetal bovine serum (Invitrogen) and a penicillin-streptomycin solution (Invitrogen) in humidified air regulated to 5% CO_2_ at 37°C. Viruses were concentrated by precipitation with 8% (wt/vol) polyethylene glycol 8000 (Sigma) and 120 mM NaCl and then purified by successive centrifugations employing a 22% (wt/wt) sucrose cushion and a 10 to 35% (wt/vol) potassium tartrate-glycerol gradient using an ultracentrifuge (Beckman) (43). Purified viruses were stored in phosphate-buffered saline (PBS) in the presence of 20% glycerol at −20°C. Infectious viruses were quantified using the focus immunoassay titration method and expressed as focus-forming units (FFU) per milliliter (53).

**TABLE 1:** Viruses employed in this study

DENV serotype	Infection/immunization of macaques			Target of antibody binding	
	Wild-type virus	LAV^*a*^	Host	Strain	Host
1	03-0398	cD1-4pm^*b*^^,^^*c*^	Vero	Hawaii	Vero

2	03-0420	cD2-4pm^*b*^^,^^*c*^	Vero	16681	C6/36
				prE203A^*f*^	C6/36

3	C06-129	cD3-3pm^*c*^	Vero	16562	C6/36

4	02-0201-5	cD4-3pm^*d*^	Vero	1036	Vero
		cD4-(2+1)pm^*e*^		C0036/06	C6/36

a A series of recombinant viruses with the chimeric prM+E sequence from the indicated dengue virus serotype, the DENV-2 genetic background of strain 16681, and the three attenuating mutations of strain 16681 PDK-53 (71).

b Contained the prM cleavage-enhancing mutation prE/D203A at the pr-M junction (52).

c Employed for both monovalent and tetravalent immunizations.

d Employed for monovalent immunization.

e Contained two out of three attenuating mutations of strain 16681 PDK-53 and the prM cleavage-enhancing mutation; employed for tetravalent immunization.

f Strain prE203A was employed for the determination of the 2D22 epitope-blocking activity.

### Macaques.

DENV-naive macaques were screened for the absence of neutralizing antibodies against dengue viruses by the PRNT with a 50% reduction endpoint (PRNT_50_) or the focus reduction neutralization test with a 50% reduction endpoint (FRNT_50_) (51). Laboratory-reared, male rhesus macaques, which were infected with a recent DENV-1 isolate or its attenuated derivatives, were described previously (51). Captured cynomolgus macaques of both sexes were infected with recent isolates representing all four serotypes at a dose of 1 × 10^5^ FFU administered subcutaneously. For the monovalent prime-boost immunization, cynomolgus macaques were infected with live-attenuated recombinant strains and then boosted twice at monthly intervals with virus-like particles of the same serotype (46). In an exceptional DENV-4-related study, cynomolgus macaques were boosted twice at monthly intervals with virus-like particles beginning 210 days after the priming DENV-4 attenuated strain injection and then challenged with a DENV-4 wild-type strain 60 days later (see Table S1 in the supplemental material). For the tetravalent immunization, a combination of live-attenuated strains was injected at one site, followed by two injections of a DNA vaccine comprising prM+E-expressing plasmids at 1-month intervals using an *in vivo* electroporator (Ichor Medical Systems, CA, USA). The ratios of male to female cynomolgus macaques were 2:1 for wild-type virus infection or monovalent immunization and 1:1 for tetravalent immunization. Blood specimens were obtained from the femoral vein under ketamine hydrochloride-induced anesthesia. The samples are listed according to the type of animal experiment (Table 2 and Table S1). Experiments in macaques received prior ethical approval from the Animal Care and Use Committee of the Armed Forces Research Institute of Medical Sciences and the Animal Use Review Division of the U.S. Army Medical Research and Material Command (Thailand site 1) (PN09-06); the Animal Care and Use Committees of PT Bimana Indomedical, Bogor Agricultural University, Indonesia (P.01-14-IR, P.02-16-IR, and IPB-PRC-14-B001); and Chulalongkorn University, Thailand (Thailand site 2) (2075001 and 2075014).

**TABLE 2:** Blood samples from groups of infected and immunized macaques employed in this study

Type of macaque	DENV serotype	No. of samples^*e*^								
		Naive	Infection	Immunization						Total
				Monovalent			Tetravalent			
				LAV	VLP boost	Chal	LAV	DNA boost	Chal	
Rhesus	1^*a*^	14 (0)	4 (0)	24 (0)						42 (0)

Cynomolgus	1^*b*^	21 (9)	3 (1)	6 (2)	6 (2)		12 (6)	12 (6)		60 (26)
	2^*b*^	21 (9)	3 (1)	6 (2)	6 (2)		12 (6)	12 (6)		60 (26)
	3^*b*^	23 (7)	5 (1)	6 (0)	6 (0)		12 (6)	12 (6)		64 (20)
	4^*b*^	17 (7)	5 (1)		6 (2)	12 (4)	12 (6)	12 (6)	6 (3)	70 (29)
	4^*c*^	3 (0)	6 (0)	19 (0)	12 (0)	6 (0)				46 (0)
	4^*d*^	20 (7)	11 (1)	19 (0)	18 (2)	18 (4)	12 (6)	12 (6)	6 (3)	116 (29)

a Experiment carried out in Thailand (site 1).

b Experiment carried out in Indonesia.

c Experiment carried out in Thailand (site 2).

d The combined set of DENV-4-related samples.

e Numbers in parentheses denote samples from female macaques. LAV, live-attenuated candidate vaccine strain; VLP, virus-like particle; Chal, macaques challenged with a recent DENV-4 isolate.

### Antibodies.

Murine monoclonal antibodies 3H5 and 8A1, recognizing type-specific epitopes in the receptor-binding EDIII domain of the E protein of DENV-2 and -3, respectively (54, 55), were purified from a hybridoma culture supernatant using protein G affinity chromatography (HiTrap protein A HP; GE Healthcare). Human monoclonal antibodies 1F4, 2D22, 5J7, and 5H2 are specific for quaternary structure-dependent epitopes present on mature viral particles of DENV-1, -2, -3, and -4, respectively (14, 56–58). An engineered and humanized antibody, 513, and a murine antibody, 2H12, recognize distinct common epitopes in the EDIII domain of all four dengue virus serotypes, whereas EDE-1 C10, a human antibody, recognizes the E dimer of all four dengue virus serotypes as well as Zika virus (47, 59–63). All humanized antibodies were generated from published VH and VL sequences by cloning the synthesized coding sequences into the human IgG expression vector pVitro1-IgG1 as previously described (64, 65). The resulting recombinant vectors were transiently transfected into Expi293 cells (Thermo Fisher Scientific). Secreted human antibodies were purified from the culture medium using protein A affinity chromatography (HiTrap protein A HP; GE Healthcare). The serotype specificity of the purified antibodies was confirmed by a dot immunoassay or an ELISA for all preparations. Antibodies were conjugated to horseradish peroxidase (HRP) using a conjugation kit according to the manufacturer’s protocol (KPL SureLink HRP conjugation kit; SeraCare). The conjugated antibody was titrated by direct binding to graded quantities of purified virus particles in an ELISA format to determine the appropriate amounts of antibody and virus to achieve an absorbance reading of approximately 1 U for use in the blocking assay. Murine antibody MOPC-21 (Sigma-Aldrich) was used as an irrelevant antibody control in cross-blocking experiments.

### Neutralization test.

Quantification of the virus-neutralizing antibody was performed by the PRNT_50_ according to the WHO protocol (66). For screening, the FRNT_50_ (51) was performed in the same manner as the PRNT_50_. When no neutralizing activity was detected at the first dilution of 1:10, an arbitrary value of 5 was used in the statistical analysis.

### Blockade-of-binding ELISA.

Aliquots of stored viruses were thawed, diluted in PBS, and mixed thoroughly by multiple rounds of pulse vortexing before a pretitrated amount was applied to the wells of a 96-well microtiter plate (MaxiSorp; Nunc) overnight at 4°C. Unbound viruses were removed, and nonspecific binding sites were blocked with 1% bovine serum albumin (BSA) (heat shock fraction, pH 7; Sigma-Aldrich) in PBS at room temperature for 1 h. Successful dilution, mixing, and adsorption of viruses to the wells were achieved when the application of a pretitrated amount of enzyme-conjugated monoclonal antibody and the chromogenic substrate showed a uniform distribution of 1 U of the optical density (450 nm) reading with minimal variation across the plate. To measure the epitope-blocking activity in blood samples, serially diluted serum or plasma samples in PBS were applied to virus-coated/BSA-treated wells in parallel with graded concentrations of an unconjugated monoclonal antibody at room temperature (or at 37°C in the cases of 5J7 and 8A1) for 1.5 h. As controls, triplicate uncoated/BSA-treated wells (background control) and virus-coated/BSA-treated wells without serum or plasma addition (binding control) were present in all plates. After the removal of the unbound serum/plasma by washing the wells three times with 0.05% Tween 20 in PBS, the corresponding HRP-conjugated monoclonal antibody was added to all wells at room temperature for 1 h. After antibody binding, the plates were washed three times with PBS, and a substrate mixture containing H_2_O_2_ and 3,3′,5,5′-tetramethylbenzidine (TMB) (1-step ultra TMB-ELISA; Thermo Scientific) was added. The reaction was stopped by the addition of 1 N sulfuric acid before the absorbance was measured at 450 nm using a microplate reader (Synergy HT; BioTek). For the binding control wells with a target absorbance reading of ~1, background control readings were generally <0.08.

The percent reduction in the absorbance reading compared with the binding control well was determined for each experimental well. A reference curve was generated from the percent reduction of graded concentrations of unconjugated monoclonal antibodies. The relative concentration of the epitope-blocking activity of each sample was determined by interpolation of the reference curve using the percent reduction at the first dilution (1:40 in all experiments in this study). For samples with a percent reduction exceeding the maximum level in the reference curve, the relative concentration was calculated from the dilution at which a 50% reduction in the absorbance could be detected by interpolation; this dilution was then used to multiply the concentration of unconjugated antibody that resulted in a 50% reduction in the reference curve to obtain the relative concentration of epitope-blocking activity for the sample.

For the variant epitope-blocking assay, virus-coated wells were treated with 15% fetal bovine calf serum (Gibco, Invitrogen) in PBS, and serially diluted serum or plasma samples were mixed with the HRP-conjugated 2D22 antibody, incubated for 1 h at room temperature, and then applied onto virus-coated/calf serum-treated wells. Monoclonal antibodies were tested after purification (as a blocking agent) and conjugation with HRP (as a binding agent) in an autologous-blocking and cross-blocking manner. Representative results for the reference curves are shown in Fig. S1.

### EDIII-binding and virus particle-binding ELISAs.

Measurements of DENV-2 EDIII-binding and particle-binding antibodies by ELISAs were performed as described previously (53). Briefly, the recombinant EDIII domain (residues 295 to 401) of strain 16681 was expressed in Escherichia coli from a pET3c vector (Novagen) (62). Inclusion bodies were denatured, refolded, and purified by size exclusion chromatography. Purified DENV-2 EDIII (150 ng/well), DENV-2 particles (200 ng/well), or BSA was applied to separate wells of the ELISA plate. Serum/plasma samples were diluted serially starting from 1:40. Bound antibodies were detected by using HRP-conjugated goat anti-human IgG antibody or rabbit anti-monkey IgG antibody (Sigma) and H_2_O_2_-TMB substrate. The EDIII- or particle-specific absorbance was obtained by subtracting the EDIII-binding or particle-binding absorbance from the BSA-binding absorbance. The endpoint titer (EPT) was determined as the reciprocal of the dilution that resulted in an EDIII-specific absorbance of 0.341 (representing an unusually high EDIII-specific absorbance value from a naive macaque sample) or a particle-specific absorbance of 0.300. A sample with a value lower than the endpoint value at the first dilution (1:40) was assigned an arbitrary titer of 20. A monoclonal antibody, clone 2H12, which recognizes a cryptic epitope on the DENV EDIII domain (62), was employed as a control for the comparison of coated EDIII/particles between plates. Pooled convalescent-phase plasma and pooled negative donor plasma were used as positive and negative controls, respectively.

### Statistical analysis.

The relationship between the epitope-blocking activity and the neutralizing antibody titer in blood samples drawn on day 30 after infection/immunization was assessed by the Spearman correlation coefficient (rho) using GraphPad Prism software (version 9.0.0). Samples drawn before the intervention showed a range of ELISA-derived values and, as a result of screening, a uniform absence of neutralizing activity. When baseline samples were analyzed together with those drawn at various time points after infection or immunization for each macaque, the correlation between the epitope-blocking activity and neutralizing activity was determined using analysis of covariance (ANCOVA) (67, 68), reported as the repeated-measures correlation coefficient (*r*_rm_), using the rmcorr package in the R program version 4.2.1 and R studio (69), which was also used to generate individual regression lines for graphical illustration. Rho and *r*_rm_ values were regarded as weak (0.10 to 0.39), moderate (0.40 to 0.69), strong (0.70 to 0.89), and very strong (0.90 to 1.00) correlations according to guidelines for interpreting the strength of a correlation (70). A correlation *P* value of <0.05 was considered significant.
