# Supplementary figures and images for: Blockade-of-Binding Activities toward Envelope-Associated, Type-Specific Epitopes as a Correlative Marker for Dengue Virus-Neutralizing Antibody
Source: Microbiol Spectr. 2023 Jul 6;11(4):e00918-23. doi: 10.1128/spectrum.00918-23 (PMC10433959; doi:10.1128/spectrum.00918-23)

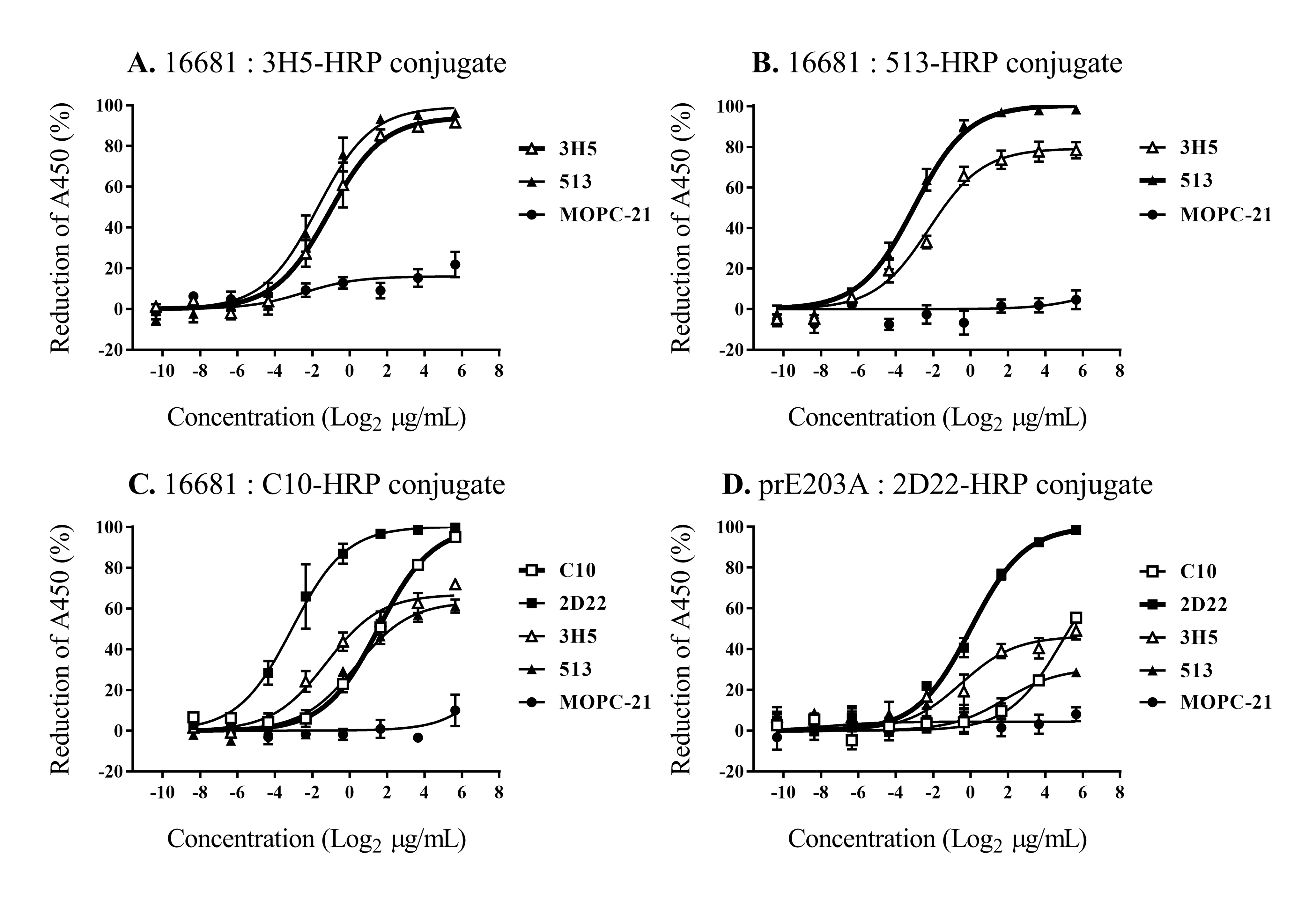

Supplement: Supplemental file 1 — Supplemental material. Download spectrum.00918-23-s0001.tif, TIF file, 0.5 MB [file spectrum.00918-23-s0001.tif]

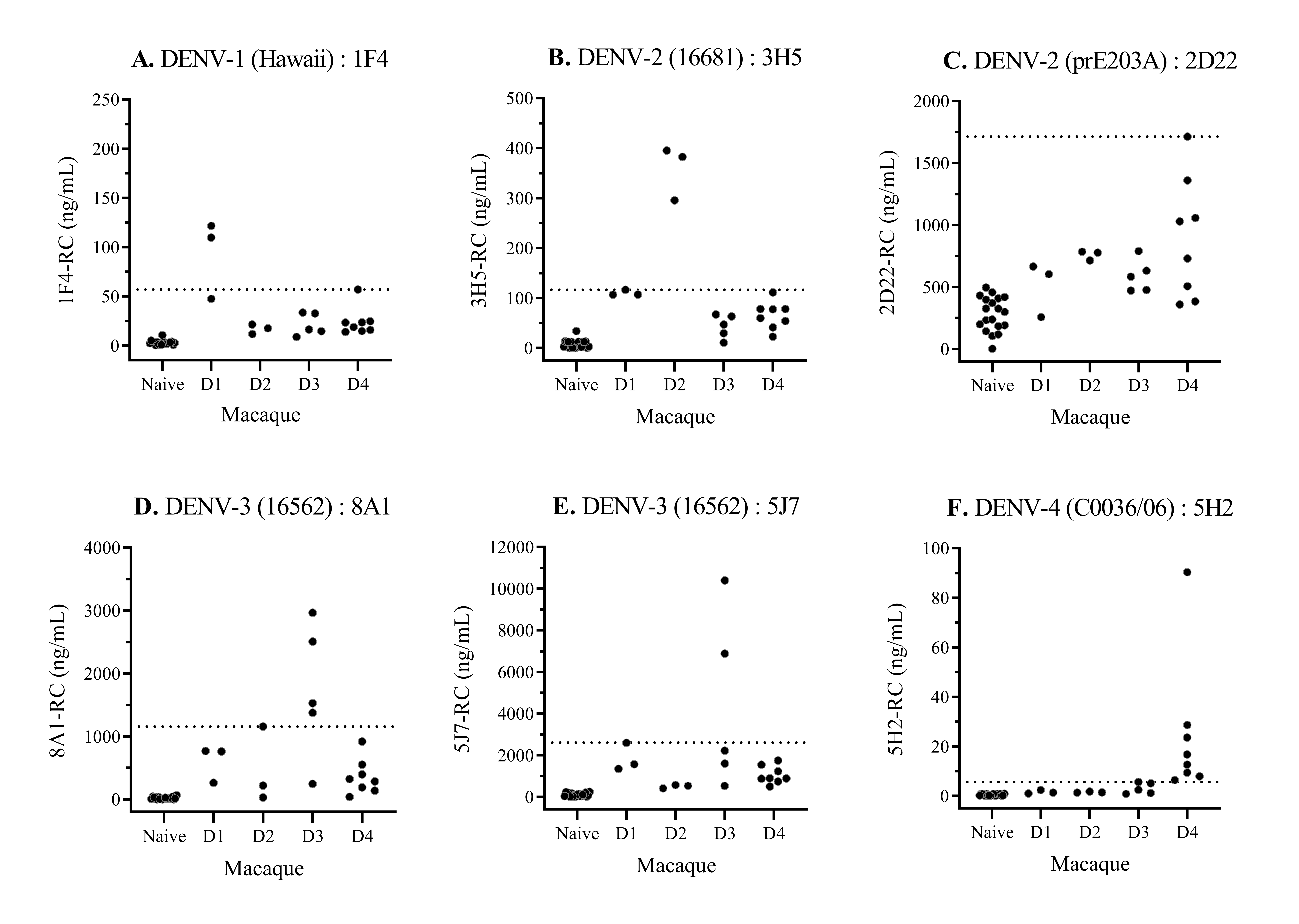

Supplement: Supplemental file 2 — Supplemental material. Download spectrum.00918-23-s0002.tif, TIF file, 1.7 MB [file spectrum.00918-23-s0002.tif]
