# Supplementary material for: Blockade-of-Binding Activities toward Envelope-Associated, Type-Specific Epitopes as a Correlative Marker for Dengue Virus-Neutralizing Antibody
Source: Microbiol Spectr. 2023 Jul 6;11(4):e00918-23. doi: 10.1128/spectrum.00918-23 (PMC10433959; doi:10.1128/spectrum.00918-23)
Supplement: Supplemental file 3 — Supplemental material. Download spectrum.00918-23-s0003.docx, DOCX file, 0.01 MB [file spectrum.00918-23-s0003.docx]

**Table S1.** Timing of blood samplings from groups of infected and immunized macaques employed in this study

| **Macaque** | **Type** | **Naive**  **(Day)** | **Infection**  **(Day)** | **Immunization (Day)** | | | | | |
| --- | --- | --- | --- | --- | --- | --- | --- | --- | --- |
|  |  |  |  | **Monovalent** | | | **Tetravalent** | | |
|  |  |  |  | LAV ^d^ | VLP boost | Chal. ^e^ | LAV ^d^ | DNA boost | Chal. ^e^ |
| Rhesus | 1^a^ | -8 | 14, 30 | 14, 30 | - | - | - | - | - |
| Cynomolgus | 1^b^ | -30 | 30 | 30 | 118 | - | 30 | 118 | - |
|  | 2^b^ | -30 | 30 | 30 | 118 | - | 30 | 118 | - |
|  | 3^b^ | -30 | 30 | 60 | 118 | - | 30 | 118 | - |
|  | 4^b^ | -30 | 30 | - | 118 | 134, 150 | 30 | 118 | 150 |
|  | 4^c^ | -10, 0 | 15, 30, 60 | 14, 30, 45, 60, 210 | 225, 240, 255,  270 | 285, 301 | - | - | - |

a, experiment was carried out in Thailand (site 1). b, experiment was carried out in Indonesia. c, experiment was carried out in Thailand (site 2). d, LAV, live, attenuated vaccine candidate strain. e, Chal., macaques were challenged with a recent DENV-4 isolate.
