# Supplementary material for: Blockade-of-Binding Activities toward Envelope-Associated, Type-Specific Epitopes as a Correlative Marker for Dengue Virus-Neutralizing Antibody
Source: Microbiol Spectr. 2023 Jul 6;11(4):e00918-23. doi: 10.1128/spectrum.00918-23 (PMC10433959; doi:10.1128/spectrum.00918-23)
Supplement: Supplemental file 4 — Supplemental material. Download spectrum.00918-23-s0004.docx, DOCX file, 0.01 MB [file spectrum.00918-23-s0004.docx]

**Table S2.** The EDIII-binding endpoint titer in plasma/sera of DENV-infected macaques.

| DENV-2 strain employed in infection/immunization | Macaque ID | End point titer ^a^ |
| --- | --- | --- |
| 03-0420 | J220211B | 20 |
|  | J080410A | 20 |
|  | J141110A | 20 |
| cD2-4pm | J030510D | 20 |
|  | J090110B | 20 |
|  | J010510B | 20 |
|  | J110410A | 20 |
|  | J230410C | 20 |
|  | J140412C | 20 |
| cD2-4pm  in a tetravalent  LAV preparation | J251010C | 20 |
|  | J090510C | 20 |
|  | J030510B | 20 |
|  | J050612A | 20 |
|  | J021210B | 20 |
|  | J181211B | 79 |
|  | J010411A | 20 |
|  | J150210B | 20 |
|  | J051010A | 20 |
|  | J170712A | 20 |
|  | J111110A | 20 |
|  | J171110D | 20 |

a, The absorbance of recombinant EDIII-coated wells was subtracted with the absorbance of corresponding BSA-coated wells, and the highest EDIII-specific absorbance among naïve samples, 0.340, was used as the endpoint. The test was performed at 1:40 dilution of plasma/sera. The sample that did not result in the specific absorbance of 0.341 or more was assigned the endpoint titer of 20.
