# Supplementary material for: Blockade-of-Binding Activities toward Envelope-Associated, Type-Specific Epitopes as a Correlative Marker for Dengue Virus-Neutralizing Antibody
Source: Microbiol Spectr. 2023 Jul 6;11(4):e00918-23. doi: 10.1128/spectrum.00918-23 (PMC10433959; doi:10.1128/spectrum.00918-23)
Supplement: Supplemental file 5 — Supplemental material. Download spectrum.00918-23-s0005.docx, DOCX file, 0.3 MB [file spectrum.00918-23-s0005.docx]

**

**

**Fig. S1 Cross-blocking Effect Among Anti-DENV-2 E Monoclonal Antibodies**

**Method** Increasing concentrations of purified monoclonal antibodies were incubated with plate-bound virus strain for one hour. Unbound antibodies were removed, and a pre-titrated amount of HRP-conjugated antibody was added for an hour and then washed off. The bound HRP-conjugated antibodies were quantified by the addition of a substrate. The blocking activity of unconjugated antibodies was calculated at each concentration as the percent reduction of the A450 from the control well (without unconjugated antibody) level of about 1 unit. Data points represent the mean and error bars represent the SEM from 3-5 independent experiments. The lines represent the nonlinear curves fitted to the data using the Sigmoidal, 4PL, X is log (concentration) function in the GraphPad Prism software version 9.0.0. The thick line indicates the blocking of the HRP-conjugated antibody by the corresponding unconjugated antibody of the same monoclonal antibody.

**Finding** The two antibodies, 3H5 and 513, which recognize the “lateral ridge” type-specific epitope (1) and the “A strand” dengue group epitope (2), respectively, on the EDIII domain of DENV-2, blocked each other’s binding to a large extent (**A** **and B**). Antibody 2D22, which recognizes distinct EDII-associated epitopes (3, 4), and an EDE-1 antibody, C10 (5), did not block these EDIII antibodies (**A and B**). Clone 2D22 efficiently blocked the binding of C10, whereas 3H5 and 513 were less active **(C)**. C10 and 3H5 displayed some blocking activity against 2D22 (**D**). At the highest concentration tested, MOPC-21, an irrelevant antibody, resulted in less than 20% blocking.

**
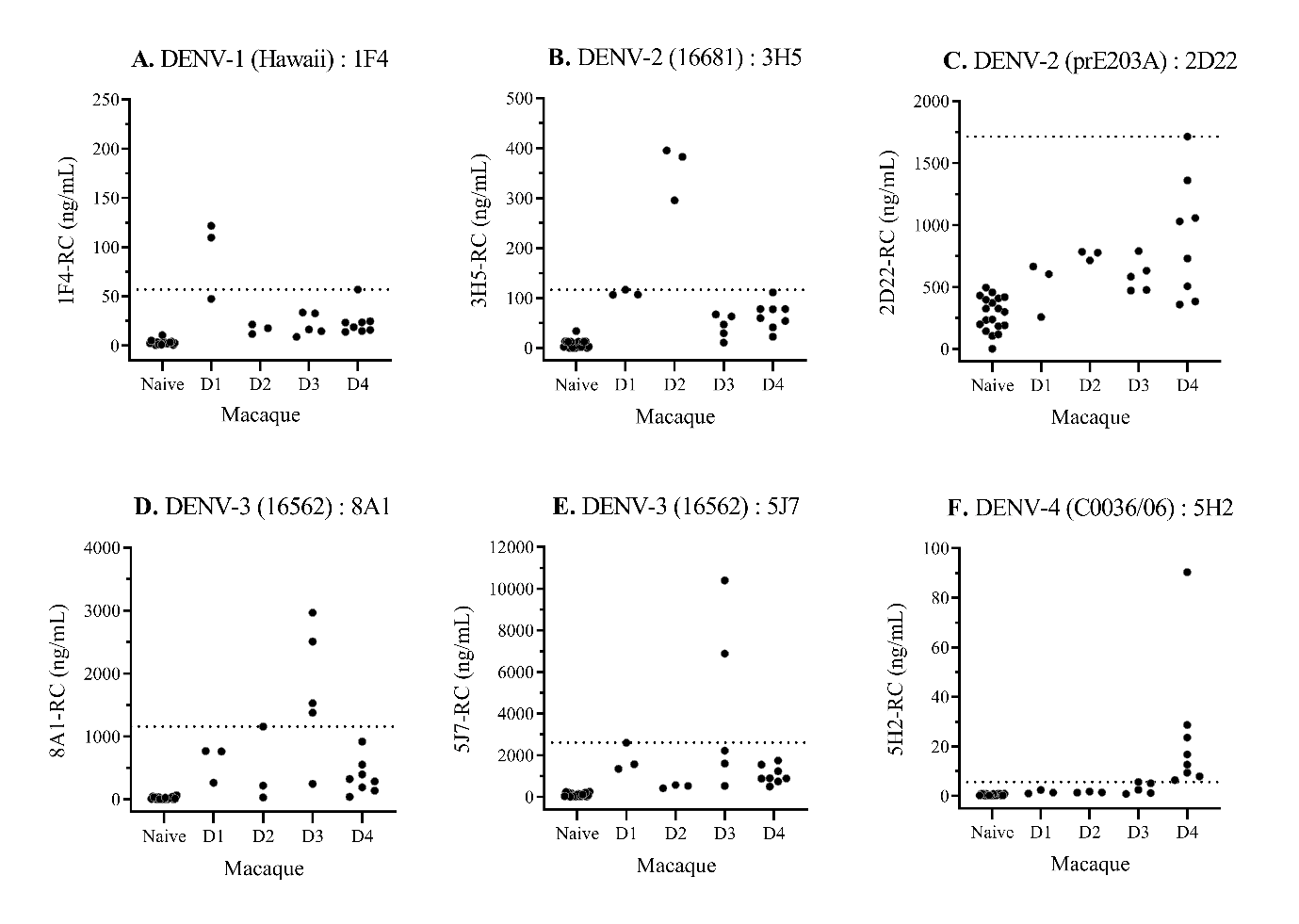
**

**Fig. S2 Interference of Type-Specific Epitope-Blocking Activity with Heterologous Cross-Reactive Antibodies**.

**Method.** Groups of macaques were infected with the indicated dengue viruses (D1, D2, D3, and D4), representing the four serotypes. Blood samples were taken before (naïve) and one month after infection and were tested in the epitope-blocking assay with six virus strains: type-specific HRP-conjugated antibody combinations (**A – F**). At the first dilution of the sample (1:40), the relative concentration of antibodies capable of blocking antibody-HRP conjugate binding to the virus was determined from a reference curve generated from the data obtained from the same plate. The dotted line represents the baseline serotype specificity, defined as the highest value of the relative concentration detected in any of the groups of macaques that were infected with viruses other than the serotype employed in each blocking test.

**Findings** (**A**). Before infection, the baseline 1F4 epitope-blocking activity was negligible. One month after infection, 1F4 epitope-blocking activity was detected at comparatively low levels in the majority (15 of 16) non-DENV-1-infected macaques whereas two out of three DENV-1-infected macaques had higher levels. Following primary infection with DENV-2, -3, or -4, heterologous cross-reactive antibodies capable of blocking the interaction between enzyme-conjugated 1F4 and the corresponding type-specific epitope on DENV-1 particles were commonly present at low levels. (**B, D, and F**). Similar results were observed with three additional type-specific monoclonal antibodies (3H5, 8A1, and 5H2) when DENV-2, -3, and -4 particles were used as targets, respectively. As in the case of 1F4, one out of the five macaques showed a low level of 8A1 epitope-blocking activity following DENV-3 infection (**C**). In contrast, comparatively high levels of 2D22 epitope-blocking activity were detected in four out of eight DENV-4-infected macaques (**C**: D4 set), and low levels of 2D22 epitope-blocking activity were found in all three DENV-2-infected macaques (**C**: D2 set). The majority (3 of 5) of macaques infected with DENV-3 had low 5J7 epitope-blocking activity (**E**: D3 set). It is recommended that 2D22 and 5J7 may be less suitable for use in blockade-of-binding assays to measure serotype-specific antibodies in the population than 3H5 and 8A1.

**SUPPLEMENTARY REFERENCES**

1. Pitcher TJ, Sarathy VV, Matsui K, Gromowski GD, Huang CY-H, Barrett ADT. 2015. Functional analysis of dengue virus (DENV) type 2 envelope protein domain 3 type-specific and DENV complex-reactive critical epitope residues. J Gen Virol 96:288-293.
2. Robinson LN, Tharakaraman K, Rowley KJ, Costa VV, Chan KR, Wong YH, Ong LC, Tan HC, Koch T, Cain D, Kirloskar R, Viswanathan K, Liew CW, Tissire H, Ramakrishnan B, Myette JR, Babcock GJ, Sasisekharan V, Alonso S, Chen J, Lescar J, Shriver Z, Ooi EE, Sasisekharan R. 2015. Structure-guided design of an anti-dengue antibody directed to a non-immunodominant epitope. Cell 162:493–504.
3. Dejnirattisai W, Wongwiwat W, Supasa S, Zhang X, Dai X, Rouvinsky A, Jumnainsong A, Edwards C, Quyen NTH, Duangchinda T, Grimes JM, Tsai W-Y, Lai C-Y, Wang W-K, Malasit P, Farrar J, Simmons CP, Zhou ZH, Rey FA, Mongkolsapaya J, Screaton GR. 2015. A new class of highly potent, broadly neutralizing antibodies isolated from viremic patients infected with dengue virus. Nat Immunol 16:170-177.
4. Barba-Spaeth G, Dejnirattisai W, Rouvinski A, Vaney MC, Medits I, Sharma A, Simon-Lorière E, Sakuntabhai A, Cao-Lormeau VM, Haouz A, England P, Stiasny K, Mongkolsapaya J, Heinz FX, Screaton GR, Rey FA. 2016. Structural basis of potent Zika–dengue virus antibody cross-neutralization. Nature 536:48-53.
5. Zhang S, Kostyuchenko VA, Ng TS, Lim XN, Ooi JSG, Lambert S, Tan TY, Widman DG, Shi J, Baric RS, Lok SM. 2016. Neutralization mechanism of a highly potent antibody against Zika virus. Nat Comm 7:13679.
